# Redox Stimulation of Human THP-1 Monocytes in Response to Cold Physical Plasma

**DOI:** 10.1155/2016/5910695

**Published:** 2015-11-15

**Authors:** Sander Bekeschus, Anke Schmidt, Lydia Bethge, Kai Masur, Thomas von Woedtke, Sybille Hasse, Kristian Wende

**Affiliations:** Leibniz Institute for Plasma Science and Technology (INP Greifswald), Felix-Hausdorff-Straße 2, 17489 Greifswald, Germany

## Abstract

In plasma medicine, cold physical plasma delivers a delicate mixture of reactive components to cells and tissues. Recent studies suggested a beneficial role of cold plasma in wound healing. Yet, the biological processes related to the redox modulation via plasma are not fully understood. We here used the monocytic cell line THP-1 as a model to test their response to cold plasma* in vitro*. Intriguingly, short term plasma treatment stimulated cell growth. Longer exposure only modestly compromised cell viability but apparently supported the growth of cells that were enlarged in size and that showed enhanced metabolic activity. A significantly increased mitochondrial content in plasma treated cells supported this notion. On THP-1 cell proteome level, we identified an increase of protein translation with key regulatory proteins being involved in redox regulation (hypoxia inducible factor 2*α*), differentiation (retinoic acid signaling and interferon inducible factors), and cell growth (Yin Yang 1). Regulation of inflammation is a key element in many chronic diseases, and we found a significantly increased expression of the anti-inflammatory heme oxygenase 1 (*HMOX1*) and of the neutrophil attractant chemokine interleukin-8 (IL-8). Together, these results foster the view that cold physical plasma modulates the redox balance and inflammatory processes in wound related cells.

## 1. Introduction

Plasma is generated by substantial energy input to a gas, thereby creating a “fourth state of matter” [1]. Plasma contains active and reactive components of many kinds including electrons, ions, and reactive oxygen and nitrogen species (ROS and RNS) as well as ultraviolet (UV), visible, and infrared radiation [2]. Apart from naturally occurring plasma, for example, lightning, fire, or* Aurelia borealis*, plasma is also created artificially. For plasma jets, such as the one used in the present study (kiNPen), the reactive components of the plasma can be delivered directly and in a controlled manner to cells and tissues without inducing thermal damage [3]. In the device (or kiNPen), the noble gas argon is excited at a central high voltage electrode (AC, several kV, ≈1 MHz). This creates nonequilibrium plasma that contains hot and reactive electrons and relatively cold argon ions in a bulk of nonionized gas atoms that govern the overall temperature [4]. The electrons and argon ions [5] in turn react with ambient oxygen or nitrogen molecules to form ROS/RNS [6]. This may be of therapeutic benefit in pathological skin conditions, as first small-scale clinical studies supported the notion that chronic wounds displayed an improved healing signature after exposure to ROS/RNS generating plasma [7–9].

Cells of the immune system are central players in the regulation of all wound healing phases [10]. During the inflammatory phase, professional phagocytes are attracted via cytokines and reactive molecules from the vasculature to the wound bed where they eradicate invading microorganisms [11]. Among them are monocytes which* in situ* differentiate into macrophages [12]. Macrophages also dominate the center of chronic ulcers and can potently generate reactive species [13]. These cells have key regulatory functions which are reflected by, for example, the phagocytosis of dead cells [14] and debris as well as their release of cytokines [15] and other wound healing-related proteins, such as heme oxygenase 1 (HO-1) [16]. In particular, interleukin-8 (IL-8) is a central molecule in initiating the inflammatory wound healing phase [17] and its secretion was previously shown to be accented by reactive species, such as hydroxyl radicals [18].

Wound healing is highly redox-controlled [19]. Almost all cells of the wound microenvironment utilize oxygen to generate reactive species, and at low concentration these species seem to be required for proper healing [20]. It is hypothesized that plasma may trigger similar responses in cells. Yet, the mechanisms and biological processes following exposure to plasma are not fully understood [21]. Accordingly, we investigated the neoplastic cell line THP-1 monocytes as a model to study the effects of a plasma-based redox modulation. We identified an increase in free thiol content, cell proliferation, and metabolic activity, arguing for a priming of these cells which was further manifested by significant changes in protein expression as analyzed by mass spectrometry based proteomic tools. We further identified an altered inflammatory signature. Our results implicate a potential significance of redox intonations by plasma that may help to further understand its impending benefit in human skin pathologies.

## 2. Materials and Methods

### 2.1. Cell Culture

THP-1 monocytes (ACC 16, DSMZ, Germany) were cultured in RPMI1640 medium (Lonza, Switzerland) containing 10% bovine calf serum (Sigma, USA), 2% glutamine (Pan-Biotech, Germany), and 1% penicillin/streptomycin (Lonza, Switzerland). For total protein expression using stable isotope labeling (SILAC), cells were labeled for at least three passages in complete RPMI containing 10% of dialyzed bovine calf serum with either both isotopically labeled arginine and lysine (^13^C_6_^15^N_4_ arginine and ^13^C_6_^15^N_2_ lysine, Cambridge Isotopes, USA) or regular amino acids.

### 2.2. Plasma Source and Treatment

The atmospheric pressure argon plasma jet* kiNPen 11* (neoplas tools GmbH, Germany) was used. It was operated with five standard liters per minute (slm) of argon gas (Air Liquide, France) and its flux was controlled using a mass flow controller (MKS instruments, Germany). The jet is equipped with a ceramic capillary (inner radius of 0.8 mm) and an inner pin-type high-frequency electrode (AC ≈ 1 MHz, several kV). Cells were indirectly plasma-treated in experiments where RNA and protein were collected for real-time PCR or mass spectrometry, respectively. Here, 5 mL of cell culture medium in a 60 mm dish was plasma-treated and was subsequently added to 1 × 10^6^ pelleted THP-1 cells. For direct plasma treatment in all other experiments, 5 mL of cell suspension (0.2 × 10^6^/mL of medium) was added to a 60 mm plastic dish (Sarstedt, Germany) and exposed to plasma. In both regimes and using a computer-controlled *xyz*-table (S-400, CNC step, Germany), the jet hovered over the sample in a meandering fashion for the indicated time length. A predetermined amount of destilled water was added after treatment to compensate evaporation. The direct and indirect approaches were previously compared and found to have similar effects. Using the same plasma source, this accounts for toxicity in human PBMC [22] and effects on metabolic activity and proliferation in feline epithelial cells [23]. Analogous results were obtained for other sources using human epithelial cells [24] as well as for inactivating bacteria [25].

### 2.3. Metabolic Activity Assay

THP-1 monocytes were plasma-treated, aliquoted in 96-well plates (Sarstedt, Germany), and incubated for 72 h at 37°C. Resazurin (Alfa Aesar, USA) was added (final concentration 200 *μ*M), and cells were incubated for 4 h. In active cells, NADH^+^ is generated during the cellular metabolism which sponsors the transformation of resazurin to its fluorescent product resorfurin [26]. The latter can be detected measuring its fluorescence at 590 nm (excitation wavelength 530 nm) using a microplate reader (M200 pro, Tecan, Austria).

### 2.4. Flow Cytometry

To assess intracellular redox changes, THP-1 monocytes were stained with 2 *μ*M CM-H_2_DCF-DA (Life Technologies, USA) prior to plasma-treatment and subsequent analysis by flow cytometry (Gallios, Beckman-Coulter, USA). Also, cells were plasma-treated, collected at various incubation times, and stained with 10 *μ*M of the ThiolTracker probe (Life Technologies) to assess the intracellular reduced thiol content. Apoptosis was investigated as previously described [27]. Briefly, cells were plasma-treated and after 24 h they were incubated in Annexin V binding buffer containing Annexin V FITC (BioLegend, USA) and 4′,6-diamidin-2-phenylindol (DAPI, Sigma, USA). Alternatively, cells were fixed in ethanol and stained with DAPI to analyze THP-1 cell cycle. To analyze the metabolic activity per cell, resorfurin fluorescence was measured, and DAPI was added prior to cell enumeration using volumetric flow cytometry (Attune, Life Technologies). Viable cell counts were related to total fluorescence obtained by the resazurin-reduction assay. Mean forward and side scatter fluorescence intensity was evaluated as well. To determine mitochondrial content, cells were stained with 0.5 *μ*M MitoTracker red FM (Life Technologies) 72 h after plasma treatment.

### 2.5. Global Protein Expression

Proteomic analysis was carried out as previously described [28]. Briefly, SILAC-labeled THP-1 cells were exposed to plasma-treated (3 min) medium and cellular protein was collected 24 h later. Proteins were fractionated using SDS gel electrophoresis and in-gel protein digestion was performed. Peptides were analyzed by nano-LC/MS (Proxeon, Denmark), and the eluent was ionized by electrospray ionization and examined using a TripleTOF 5600 (AB Sciex, USA) mass spectrometer. Data were processed using ProteinPilot 4.5 software (AB Sciex) and Ingenuity Pathway Analysis (Qiagen, USA). Between 2200 and 2700 human proteins from about 60,000 peptides were identified out of 240,000 mass spectra. Candidates were selected upon their significant involvement in pathways of metabolisms and/or redox stress as well as on statistical criteria (≥±1.5-fold expression). Additionally, data were analyzed through the use of* Ingenuity Pathway Analysis* (IPA, Qiagen) and free web based applications (*PANTHER* and* Universal Protein Resource*).

### 2.6. Real-Time PCR of* IL-8* and* HMOX1*

THP-1 cells were exposed to plasma-treated medium and incubated for 12 h or 24 h. Cells were harvested and mRNA was isolated (RNA-Mini Kit; Bio&Sell, Germany). Complementary DNA (cDNA) was generated from total RNA using transcriptor first strand cDNA synthesis kit and T4 DNA polymerase for second strand synthesis (both from Roche, Switzerland). For quantitative polymerase chain reaction, the real-time ready catalogue assay kit containing* IL-8* and* HMOX1* primers was utilized, and samples were analyzed using a LightCycler 480 II (all Roche).

### 2.7. Cytokine Detection

THP-1 cells were incubated for 24 h after plasma treatment. Supernatants were analyzed using an IL-8 (BioLegend) ELISA and a multianalyte inflammatory cytokine ELISA array (Qiagen). Sample values were normalized to control values and displayed as fold change to control.

### 2.8. Statistics

Experiments were repeated at least three times. Values were displayed as arithmetic mean. For MitoTracker and ThiolTracker fluorescence and for IL-8 and* HMOX1* expression studies,* Student*'s *t*-test was applied. For comparison in all other experiments, one-way ANOVA with* Dunnett* posttesting was utilized. Significance levels were indicated as follows: ^*∗*^ *α* = 0.05, ^*∗∗*^ *α* = 0.01, and ^*∗∗∗*^ *α* = 0.001.

## 3. Results and Discussion

### 3.1. Plasma Altered the Cellular Redox State without Major Apoptosis Induction

**Figure 1:**
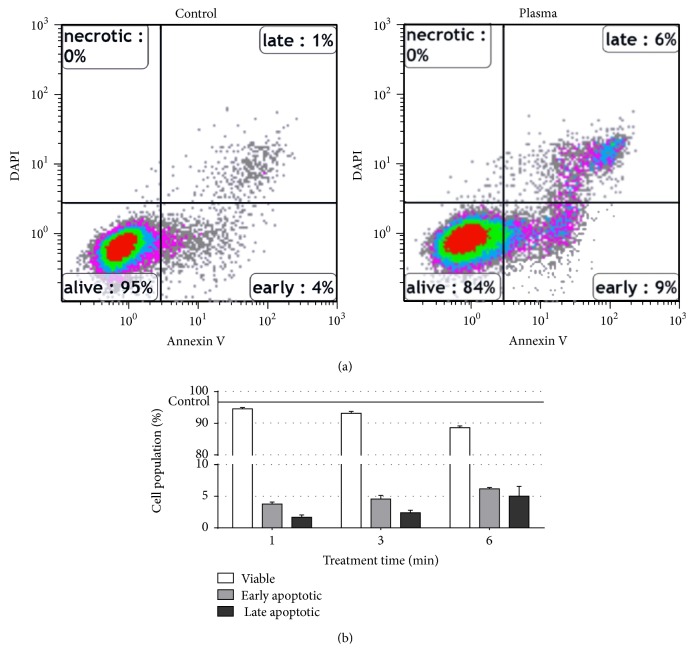
Analysis of THP-1 cell apoptosis. THP-1 monocytes were plasma-treated (6 min). Percentages of viable as well as early and late apoptotic cells were analyzed (a) and quantitated (b) by flow cytometry using Annexin V and DAPI. “Ctrl” represents percent of viable cells in untreated samples; percentages of viable and early and late apoptotic cells add up to 100. Data are shown as one representative (b) or mean + S.E. (b) of five independent experiments.

**Figure 2:**
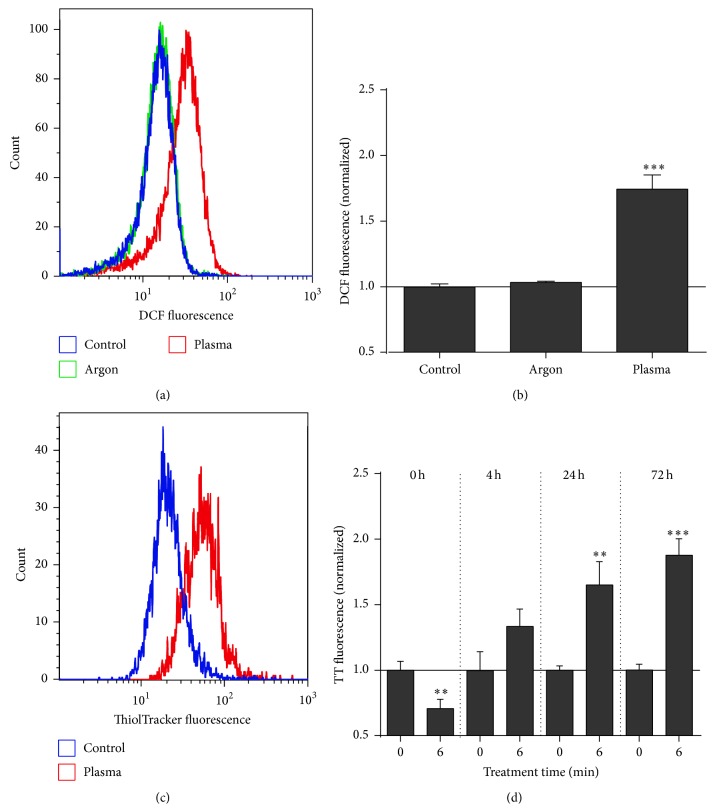
Analysis of redox changes in THP-1 cells. THP-1 cells were stained with CM-H_2_DCF-DA and plasma-treated (3 min) to evaluate intracellular redox changes by flow cytometry (a, b). THP-1 cells were plasma-treated, and cells were stained at different time point with ThiolTracker dye to examine their total reduced thiol content ((c), shown for 72 h) by flow cytometry (d). Data are shown as one representative (a, c) or mean + S.E. (b, d) of three independent experiments.

In this study, we investigated whether exposure to cold physical plasma affected THP-1 cell viability, metabolic activity, and function. THP-1 cell apoptosis was studied (Figure 1(a)), and viability was compromised only to a minor extent (Figure 1(b)). This is in line with peripheral blood monocyte viability being only marginally affected by plasma [27]. Yet, plasma did induce changes in the cellular redox state. Immediately following treatment, THP-1 cells showed a significant (*P* < 0.001) increase in DCF fluorescence (Figures 2(a) and 2(b)). Although DCF is regarded as a general redox probe, it is highly sensitive to hydroxyl radicals [29]. These may have been formed in the Fenton reaction of plasma-derived hydrogen peroxide [30] with metals being inevitably present in biological systems [31]. We asked next whether plasma-derived reactive species also affected the cells' antioxidative defense. Here, glutathione is a central molecule which constitutes and regenerates the majority of free thiols in cells to maintain redox homeostasis [32]. The intracellular free thiol content was significantly (*P* < 0.01) decreased immediately following exposure to plasma whereas thiol levels were substantially elevated hours and days after plasma treatment (Figures 2(c) and 2(d)). This argues for a swiftly augmented redox defense in response to plasma with only little induction of apoptosis. This may be explained by THP-1 cells being derived from an acute monocytic leukemia (AML), and an excessive constitutive ROS production combined with an impaired p38-MAPK and ROS-mediated apoptosis signaling was found in AMLs of oncological patients [33]. Moreover, GSH decrease was also followed by a rebound increase as an adaptive response to oxidative stress in epithelial cells [34], underlining our results, and suggested an adaptation in THP-1 cells following plasma. We subsequently investigated alterations in the cells' morphology and metabolic activity after plasma treatment.

### 3.2. Plasma Enhanced Cell Size and Stimulated Cellular Proliferation

**Figure 3:**
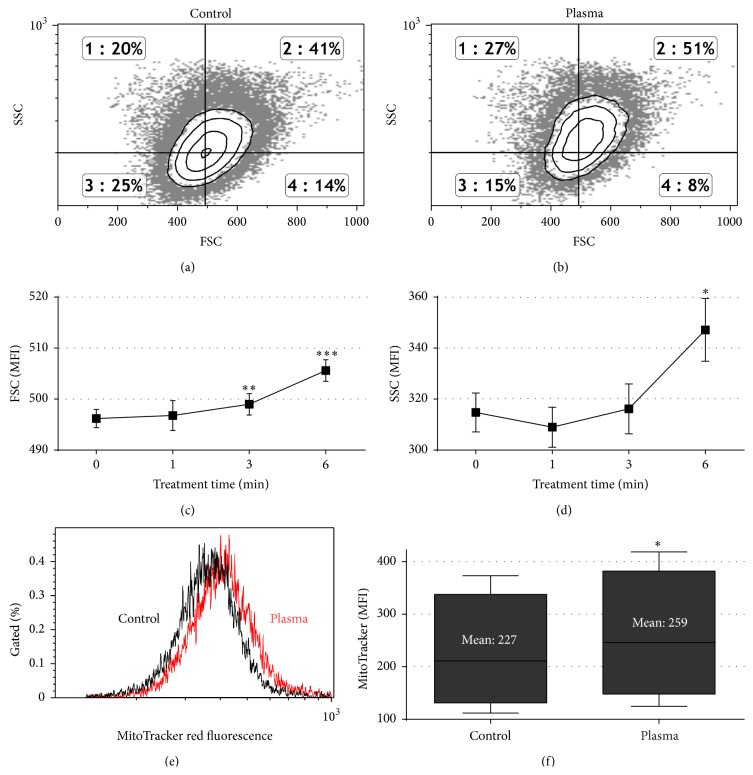
Analysis of THP-1 cell size and mitochondrial content. THP-1 monocytes were plasma-treated. After 72 h, forward (FS) and side scatter (SSC) distribution (a, b) and mean fluorescence intensity (MFI) thereof (c, d) were analyzed in viable cells using flow cytometry. MitoTracker red fluorescence indicative of total mitochondrial content was assessed in viable cells (f) 72 h after plasma treatment. Data are shown as one representative (a, b, e), mean + S.E. (c, d), or boxplot (5–95 percentile, (f)) of three independent experiments.

**Figure 4:**
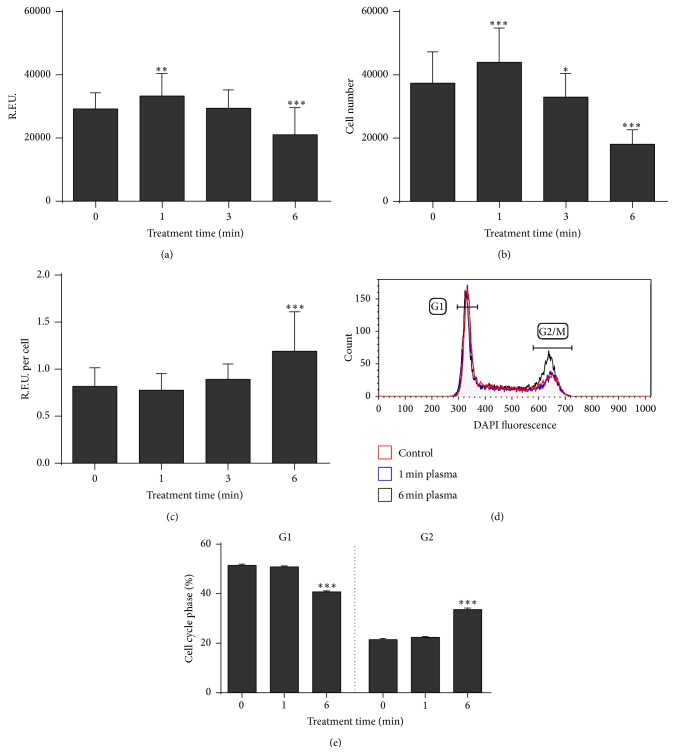
Analysis of total THP-1 cell metabolism and number. THP-1 monocytes were plasma-treated, and after 72 h the total metabolic activity was assessed in a microplate reader using the resazurin assay (a). Viable cell numbers were determined of the same samples (b), and the metabolic activity per cell was calculated (c). Twenty-four hours after plasma treatment, cells were subjected to cell cycle analysis (d) and quantitatively compared to each other (e). Data are shown as one representative (d) or mean + S.E. (a, b, c, and e) of three independent experiments.

Compared to control cells (Figure 3(a)), plasma-treated cells (Figure 3(b)) displayed a small but significant enhancement of mean forward scatter (attributed to the cells' size, Figure 3(c)) and side scatter (attributed to the cells' granularity and membrane irregularity, Figure 3(d)). This correlated with mitochondrial content (Figure 3(e)) that was significantly increased in plasma-treated cells as well (Figure 3(f)), possibly pointing to an altered metabolic activity. Indeed, we found a significant elevation (*P* < 0.003) of cellular respiration for short exposure to plasma (1 min). By contrast, total metabolic activity was significantly (*P* < 0.001) decreased after extended (6 min) plasma treatment (Figure 4(a)). The same cells that were subjected to the assessment of metabolic activity were subsequently analyzed by volumetric flow cytometry and viable cells were counted. Again, we found a significant (*P* < 0.001) increase in cell numbers for short exposure times and a significant decrease (*P* < 0.001) for longer exposure times to plasma (Figure 4(b)). Based on these data, we calculated the metabolic activity on a per-cell basis by combination of the total fluorescence divided by the total number of viable cells (Figure 4(c)). For short exposure times there was no difference compared to untreated controls. On the contrary, the per-cell metabolic activity was significantly (*P* < 0.001) enhanced in cells that were exposed to plasma for 6 min. Supplementation of fresh media during the incubation time yielded similar results and hence the elevated metabolism was not a consequence of nutrient limitation in control cells (data not shown). The decrease in total cell numbers (Figure 4(b)) was reflected by a significant (*P* < 0.001) G2 arrest (Figures 4(d) and 4(e)) 24 h but not 72 h (data not shown) after plasma treatment.

These results suggest that exposure to plasma may have stimulated THP-1 cells, eventually leading to enhanced proliferation for short exposure times. Reactive species delivered to the cells via the plasma altered the intracellular redox state and consequently stimulated growth. This may be reflected by the finding of others who suggested a role of redox changes in, for example, thioredoxin or Id3 in growth stimulation [35, 36]. It was reported that redox modifications in THP-1 monocytes lead to macrophage differentiation [37] which may account for the increase observed in cell size (Figure 3(c)) and metabolic activity (Figure 4(c)). Indeed, THP-1 cell activations via, for example, mitogens increase cell size as well [38]. Yet, mitogen-activated and THP-1 cell-derived macrophages display a distinct surface marker signature (e.g., elevated expression of CD33, CD45, CD49b, CD49d, CD81, and CD141) which was not present on plasma-treated THP-1 cells (data not shown). This argues against a role of plasma in induction of THP-1 cell differentiation. Oxidative stress was also shown to support an accumulation of THP-1 cells in the G2 phase of the cell cycle [39] which supports our findings (Figure 4(e)) and may explain the lower proliferation found after prolonged exposure to plasma (Figure 3(b)). A linkage between the enhanced metabolic activity and the elevated mitochondrial content (Figure 3(f)) is unlikely due to the monocytes' glycolytic nature [40]. It was suggested that monocytes utilize mitochondria to generate ROS for signaling purposes and not mainly for ATP generation [41]. This would imply a combined extrinsic (plasma) and intrinsic (mitochondria) ROS generation, possibly affecting redox signaling and/or other biological responses observed in this study. Finally, growth promotion of plasma-treated cells may be a consequence of elevated GSH levels (Figure 2(d)) protecting from ROS generated during proliferation [42].

### 3.3. Global Protein Expression Screening

Next, proteome analysis was carried out to further elucidate the THP-1 cell protein response to plasma. In plasma-treated THP-1 monocytes, total protein expression was increased by 1.05-fold, paralleling the observed increase in cell size (Figure 3). An increase of protein translation was also suggested by the finding that the dominant pathways regulated after plasma involved ribosomal protein translation (eIF2 signaling and eIF4/p706K signaling) [43]. Simultaneously, cellular protein degradation was elevated, indicated by an increased expression of several key members of the ubiquitin/proteasome pathway, such as SUGT1 and STUB1 (Table 1) [44, 45].

**Figure 5:**
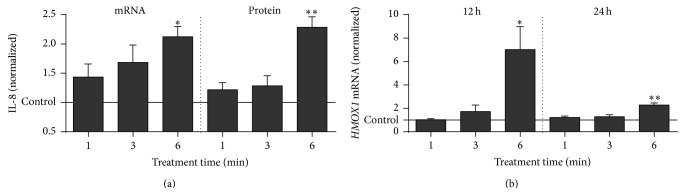
Analysis of THP-1 cell IL-8 and* HMOX1* expression. THP-1 monocytes were plasma-treated, and after 24 h* IL-8* expression and its secretion were analyzed by real-time PCR and ELISA, respectively. Expression of* HMOX1* was examined 12 h and 24 h after treatment using real-time PCR (b). Data are presented as mean + S.E. of three independent experiments.

These observations point to an anticipated protein stress response triggered by plasma and subsequent changes in the THP-1 monocyte redox state. This notion is further supported by the finding that the endothelial PAS domain-containing protein 1 (EPAS1, also known as hypoxia inducible factor-2*α*) was highly regulated after plasma (Table 1). Like HO-1, EPAS1 is central in the maintenance of redox homeostasis and is moreover linked to superoxide dismutase (SOD) activity [46]. After plasma treatment, we found a decrease in mitochondrial-resident SOD (SOD2, Table 1). Although SOD2 is a protector against oxidative stress, previous work had found downregulation during the physiological wound healing process in rats [47]. Along with our findings, decreased levels were also reported in response to sublethal oxidative stress in neuronal cells [48]. In oxidatively challenged rats, Yin Yang 1 (YY1), a zinc finger transcription factor, was strongly increased [49] which correlates with our results (Table 1). YY1 is central in cell cycle progression and resistance to apoptotic stimuli [50] and its upregulation in macrophages is associated with an increased expression of cyclooxygenase 2 (COX2) [51]. COX2 is a hallmark of tissue injury and inflammation. Thus, the increase of YY1 expression after plasma may represent the perception of a danger stimulus by THP-1 cells. This may be reflected by changes in the inflammatory signature (Figure 5) and the decrease of interferon regulatory factor 8 expression (IRF8, Table 1) which may indicate the effort to control interleukin production and overshooting cell activation [52, 53]. YY1 also controls respiratory chain expression and mitochondrial activity [54, 55] and may have a role in the modulation of the metabolic activity observed (Figure 4(c)).

However, in contrast to human keratinocytes [56], oxidative stress response like nuclear factor erythroid 2-related factor (Nrf2) regulated response was not observed as indicated by unchanged NAD(P)H dehydrogenase (quinone) 1 (NQO1) or carbonyl reductase (NADPH) 1 (CBR1) expression (data not shown). Instead, retinoic acid (RA) signaling was activated as shown by cellular retinoic acid binding protein 1 (CRABP1) expression (Table 1). Corroborating our findings, RA signaling in monocytes has been associated with a slowdown of cell cycle progression and a stimulation of cell differentiation [57, 58]. Additionally, RA signaling plays a role in the oxidative stress response and CRABP1 expression may reflect a reaction towards the reduced SOD2 levels [59].

We identified YY1 to be strongly controlled by plasma. In the human T cell line Jurkat, the IFN-*γ* cytokine promoter is under control of YY1 [60]. Malondialdehyde-induced ROS stress was found to highly upregulate YY1 expression in Jurkat cells which was subsequently associated with a marked increase of* IL-8* mRNA and its release [61]. Strikingly, YY1 is positively regulated by products of HO-1, such as carbon monoxide [62], and* HMOX1* upregulation was previously found in plasma-treated keratinocytes [56]. Both molecules are associated with inflammation and we asked next whether their expression was affected following exposure to plasma.

### 3.4. Plasma Treatment Induced Expression of IL-8 and* HMOX1*

In the inflammatory phase of wound healing, the main function of the proinflammatory chemokine IL-8 (CXCL8) is the attraction of phagocytes from the blood to the site of injury [63]. Exposure of THP-1 monocytes to plasma led to the presence of higher copy numbers of* IL-8 *mRNA (Figure 5(a)). This correlated with an increase in secretion of IL-8 (Figure 5(b)) while no release or no change in the release of other inflammation-associated molecules (IL-1*β*, IL-6, IL-10, IL-17A, IL-22, GM-CSF, INF*γ*, TNF*α*, and TGF*β*) could be observed (data not shown). IL-8 is also secreted by primary monocytes and macrophages but is not associated with autocrine effects on these cells [64], making a contribution of this molecule to other results of this study unlikely. Substantiating our results, antioxidants were found to decrease steady state ROS and IL-8 levels [65] while increased ROS trigger IL-8 expression in primary monocytes/macrophages [66].

Next to IL-8,* HMOX1* was significantly upregulated after exposure to plasma (Figure 5(b)). The myeloid cell-specific enzyme HO-1 protects against the cytotoxicity of oxidative stress and mediates immunomodulatory and anti-inflammatory properties [67] via the oxidation of free heme under generation of the products carbon monoxide, biliverdin, and iron [68]. It is upregulated during oxidative stress [69] and hypothesized to be central in the protection and homeostatic reestablishment in numerous pathological conditions [70]. Ferritin, a scavenger of free iron, is coinduced with* HMOX1* [71], which may provide a beneficial side effect via, for example, reduced hydroxyl radical generation through the Fenton reaction. Importantly, HO-1 expression was shown to increase the number of mitochondria and total metabolic activity in cardiac cells [72] which corroborates our findings in THP-1 monocytes (Figures 3(f) and 4(c)).

### 3.5. Study Limitations

Following exposure to cold physical plasma, the widely recognized cell line THP-1 monocytes were used in this study to investigate their biological responses. This includes the identification of their proteomic profile using SILAC mass spectrometry which requires proliferating cells. Yet, owing to the altered ROS-signaling in THP-1 cells [33], the findings identified in this work may not fully represent the redox response in other cell types following exposure to plasma. Moreover, studying the neoplastic THP-1 cells may provide an only imperfect model to mimic responses of wound-resident monocytes/macrophages to plasma. This especially accounts for the changes observed in metabolic activity and cell proliferation as macrophages at the wound site derive from monocyte differentiation rather than cell division [73].

## 4. Summary

Via generation of ROS/RNS, treatment with cold physical plasma may be beneficial in redox-related diseases, such as impaired wound healing. Using THP-1 monocytes as a model, we investigated the oxidative challenge provided by the plasma to these cells. Although plasma induced changes in the intracellular redox status, it only modestly compromised their viability. Short plasma treatment stimulated THP-1 monocyte growth while longer exposure increased cell size, mitochondrial content, and metabolic activity. Global protein expression analysis revealed an increase in protein synthesis, degradation, and folding processes indicative of both plasma-mediated protein stress response and changes in protein expression pattern. A moderate oxidative stress response was detected, yet not via Nrf2 signaling. Changes in RA signaling, YY1, and IRF8 expression suggested the activation of cell differentiation events. At long exposure times, plasma upregulated the expression of IL-8 (but not other inflammatory cytokines) and* HMOX1* which are both involved in inflammatory processes, such as wounds. Altogether, THP-1 monocytes mounted a distinct response to plasma which was manifested by alterations of their metabolic activity and inflammatory potential. Our results exemplify the delicate balance of cellular redox control and suggest a role of low-dose redox modulation in wound-related cells which is aimed at being triggered by cold plasma in redox-based diseases.

## Figures and Tables

**Table 1:** Differential expression of proteins complexed in metabolic and redox processes after plasma treatment. Fold regulation of relevant candidate protein expression identified from global proteomic profiling of THP-1 cells and 24 h after exposure to plasma-treated medium. Data are presented as mean and range of three independent experiments.

Short name/ID	Fold change	Pathway/task
EPAS1 (Q99814)	+4.0 ± 0.7	Transcription factor
YY1 (P25490)	+3.0 ± 0.3	Cell cycle control
CRABP1 (P29762)	+1.9 ± 0.2	Retinoic acid signaling
SUGT1 (Q9Y2Z0)	+1.9 ± 0.3	Protein ubiquitination
STUB1 (Q9UNE7)	+1.7 ± 0.2	Protein ubiquitination
SOD2 (P04179)	−1.5 ± 0.1	Redox balancer
IRF8 (Q02556)	−1.6 ± 0.2	Interferon signaling

## Abbreviations

IL-8: Interleukin-8
*HMOX1*, HO-1: Heme oxygenase 1
YY1: Yin Yang 1
SOD: Superoxide dismutase.
